# The Influence of Local Strain Distribution on the Effective Electrical Resistance of Carbon Black Filled Natural Rubber

**DOI:** 10.3390/polym13152411

**Published:** 2021-07-22

**Authors:** E. Harea, S. Datta, M. Stěnička, J. Maloch, R. Stoček

**Affiliations:** Centre of Polymer Systems, Tomas Bata University in Zlín, Tř. Tomáše Bati 5678, 76001 Zlín, Czech Republic; sdatta@utb.cz (S.D.); stenicka@utb.cz (M.S.); maloch@utb.cz (J.M.); stocek@utb.cz (R.S.)

**Keywords:** effective electrical resistance, elastomer sensors, natural rubber, local strain, conductive filler, digital image correlation

## Abstract

A monotonous relation between strain and measured electric resistance is highly appreciated in stretchable elastomer sensors. In real-life application the voids or technological holes of strained samples often induce non-homogeneous local strain. The present article focused on studying the effect of non-homogeneous local strain on measured direct current (DC) effective electric resistance (EER) on samples of natural rubber (NR), reinforced with 50, 60 and 70 phr of carbon black (CB). Samples were imparted geometrical inhomogeneities to obtain varied local strains. The resulting strain distribution was analyzed using Digital Image Correlation (DIC). EER exhibited a well-detectable influence of locations of inhomogeneities. Expectedly, the EER globally decreased with an increase in CB loading, but showed a steady increase as a function of strain for 50 and 60 phr over the complete testing protocol. Interestingly, for 70 phr of CB, under the same testing conditions, an alternating trend in EER was encountered. This newly observed behavior was explained through a novel hypothesis—“current propagation mode switching phenomenon”. Finally, experimentally measured EERs were compared with the calculated ones, obtained by summing the global current flow through a diversity of strain dependent resistive domains.

## 1. Introduction

### 1.1. Background

The change in electrical resistance of filled rubbers under mechanical stimulus opens a large number of possibilities for practical applications. The ability of cured rubber composites to deform enormously without visible failure is highly appreciated in stretchable sensors. Such sensors, prepared by blending an insulating rubber matrix with conductive fillers have a great perspective for industrial production [1]. These composites are also promising materials for transducers and flexible electrodes due to their conductivity, even in a deformed state [2,3,4].

Carbon black (CB) reinforced rubber compound consists of two interpenetrated phases with very different electrical properties: rubber forms a resistive network while CB produces a conductive network [5]. As such, a two-phase compound has an electrical conductivity dependent on both the phases.

Generally, the effective conductivity/resistivity of inhomogeneous materials was a subject of serious research for various materials and their applications. Conventionally all these studies can be divided into two groups: (1) involving the effective medium theory (EMT), averaging the multiple values of the constituents [6] and (2) focused on the calculation of equivalent resistor network (ERN) [7]

The effective electrical conductivity/resistivity was found to be dependent on relative amount of constituent phases, conductivity of phases and their distribution [7,8]. The attempt to discretize the phases in material by their geometry [7] had its advantage in reducing the complexity of EER calculation to solving the Kirchhoff equations [9] for the currents in conductive network. The suggested discretization involves substitution of resistance of one phase domain by a series of resistors, linking the neighboring domains.

Over the continuously enlarging number of investigations on highly deformable sensors, based on reinforced elastomers, the influence of strain-induced local deformation on EER has rarely been discussed.

The macroscopic geometrical inhomogeneity (e.g., cavity, voids, cracks) naturally present in rubber materials tend to form surrounding macroscopic domains with particular local strain distribution. Moreover, the intentionally produced geometrical inhomogeneity in a rubber product, which is required for its proper functioning, concentrates strain in the vicinity of the inhomogeneity during service. Therefore, the local stress–strain behavior around the inhomogeneity is close to intrinsic strength, causing crack growth initiation, whereas future loading leads to its propagation up to total failure [10]. On the other hand, the geometry of the inhomogeneity and global loading conditions are the reason for local multiaxial deformation leading to re-arrangement of the filler network at the affected location [11,12]. Thus, an influence on EER behavior is expected objectively.

Choice of composite, based on CB-filled natural rubber (NR) for the present study was dictated by several reasons:

Natural rubber (NR) is a strategic industrial raw material for manufacturing a wide variety of products, due to its very high strains and the respective ultimate strength before total rupture compared to other synthetic rubbers. NR becomes ‘‘self-reinforcing’’ at high strain level and simultaneously the mechanical properties increase. In most applications of NR, fillers are added to increase modulus, toughness and wear resistance (e.g., [13]), whereas for the rubber products with antistatic properties, the carbon black (CB) mostly is the applied conductive material along with its reinforcing properties.

The morphology and properties of filled rubber are greatly influenced by filler networking [14,15]. When a certain concentration is attained, the filler forms a continuous network that can be described in the frame of percolation or cluster, known as the cluster aggregation theory [16]. The long-range connectivity of conductive fillers can cause significant field intensification in the rubber matrix, and greatly enhance the dielectric behavior and thus the overall permittivity of the rubber matrix [17,18]. Due to the high conductivity of the CB particles, the electric field is most significantly concentrated in the small gap between two connected clusters separated by an individual distance. Thus, the electric field between two clusters changes dramatically with the distance between the interconnecting clusters, based on the ability of the electrons to overcome the gaps by tunneling effect or trap-assisted tunneling effect [19].

### 1.2. Research Approach

The experimental investigation for determination of change in electric response of cured rubber in dependence on strain generally can be done under a simple uniaxial tensile loading using the samples of strip geometry. Due to the application of a thin sample, in which the thickness significantly is lower than the length or even the width, the deformation in the direction of the thickness can fully be neglected [20,21,22].

The aim of the presented work was to perform an experimental investigation of EER of deformed rubber samples of an identical rectangular shape with implemented annular geometrical inhomogeneity, located differently across the orthogonal axes of sample to the main strain. The samples based on natural rubber (NR) reinforced with CB far above the percolation threshold (50, 60 and 70 phr), were prepared and subjected to tensile loading up to strain 26.7%. In the case of NR, the dedicated strain is still far below the strain values in which strain induced crystallization (SIC) appears [23,24]. The deformation of the sample has been monitored and the strain distribution over the complete sample surface has been determined by the Digital Image Correlation (DIC) system. The DC EER behavior has been measured simultaneously during the loading. The DC measurements was implemented taking into account the well-known facts that the current density distribution driven by the alternating current (AC) is often not uniform throughout the cross-section of any conductor because of the skin and proximity effects [25]. Finally, the proposal for numerical calculation of EER related to deformation of samples in dependence on varied geometrical inhomogeneity and the corresponding local strain distortion of conductive phase was introduced for the first time, in effect supporting the novelty of the work.

## 2. Materials and Methods

### 2.1. Rubber Formulation

The complete formulation of rubber used within this study is listed in Table 1. Natural rubber was supplied by the Astlett Rubber Inc. (Astlett Rubber Inc., Oakville, ON, Canada) (type SMR 20 CV/BP1). Sulfur used as the curing agent, zinc oxide (ZnO) and stearic acid used as activators were supplied by Sigma-Aldrich^®^. The reinforcing filler used in all the compounds was high abrasion furnace (HAF)–N330 carbon black (CB) supplied by Cabot Corporation, Boston, MA, USA. Moreover, Naphthenic oil NYTEX^®^ (Nynas AB, Nynashamn, Sweden) was used as a plasticizer and CBS (N–cyclohexyl–2-benzothiazolesulfenamide), was employed as the curing accelerator.

### 2.2. Rubber Compounding and Samples Preparation

**Figure 1:**
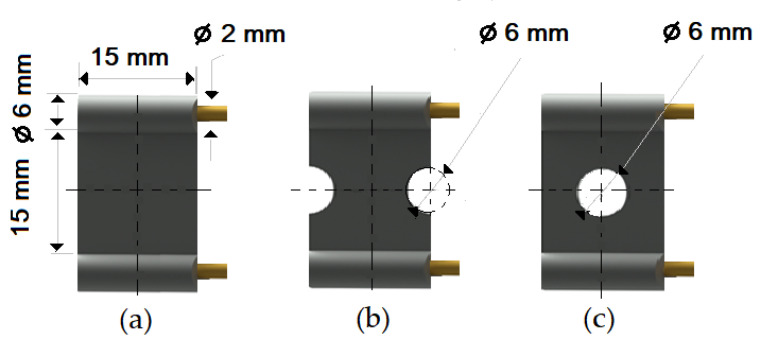
The geometry of studied samples: (**a**) basic configuration, (**b**) double side inhomogeneity, (**c**) central inhomogeneity.

The compounds were prepared in an internal mixer Brabender Plastograph (Brabender GmbH & Co., Duisburg, Germany) at 60 °C at a rotor speed of 50 rpm at a fill factor of 80%. The rubber and the compounding ingredients were successively added as follows: NR was masticated for 3 min followed by mixing of ZnO and stearic acid activators, both for 1 min successively. The filler was added in three stages alternating with the plasticizer and mixed for the next 3 min. Finally, CBS and sulfur were added and mixed for another 2 min. Thus, the total mixing time was 10 min. The optimum cure time at 160 °C for each batch was determined using a moving die rheometer (MDR 3000 MonTech, Buchen, Germany) according to ISO 3417. After 24 h conditioning at an ambient temperature of 23 °C, the compounds were molded using electrically heated hydraulic press (LabEcon, Delft, The Netherland) at 160 °C and 200 kN into samples of specific geometries defined generally with dimensions 15 × 15 × 2 mm^3^ with cylindrical shoulders of 6 mm in diameter at both ends. Each cylindrical shoulder contained a brass tube (2 mm external and 1.4 mm internal diameters) in the direction of the shoulder axis for realization of future electrical contacts. Finally, in two amongst the three different samples, shape inhomogeneities, characterized by top view of an annular circle (sample c) and two semi circles (sample b) having diameters of 6 mm were implemented. The detailed geometries of the investigated samples are shown in the Figure 1.

### 2.3. Electric Setup

**Figure 2:**
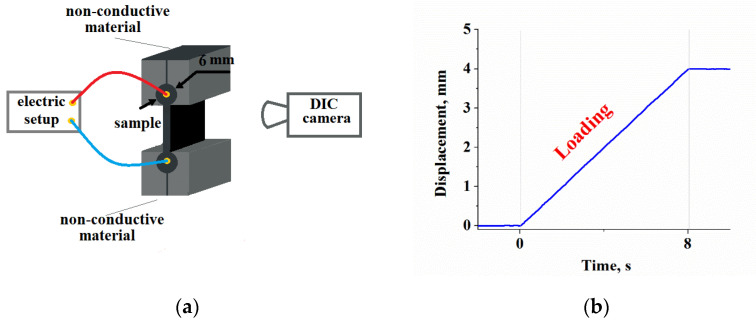
(**a**) Customized nonconductive clamps; (**b**) testing protocol.

The measurement of the DC EER in tensile mode was done in a servohydraulic testing equipment Instron 8871 (Instron, High Wycombe, UK) equipped with customized nonconductive clamps (Figure 2), which were used to fix the lateral cylindrical shoulders of the tested samples. Due to the application of cylindrical shoulders containing tubular brass contacts, the additional stress commonly induced by simple fixing system was efficiently avoided. The conductive wires were mechanically crimped into the brass tubes, avoiding meta–rubber interface overheating as encountered in the case of soldering.

The applied testing protocol, schematically visualized in Figure 2b was based on strain up to 4 mm (26.7% strain) at a constant rate of 0.5 mm/s.

**Figure 3:**
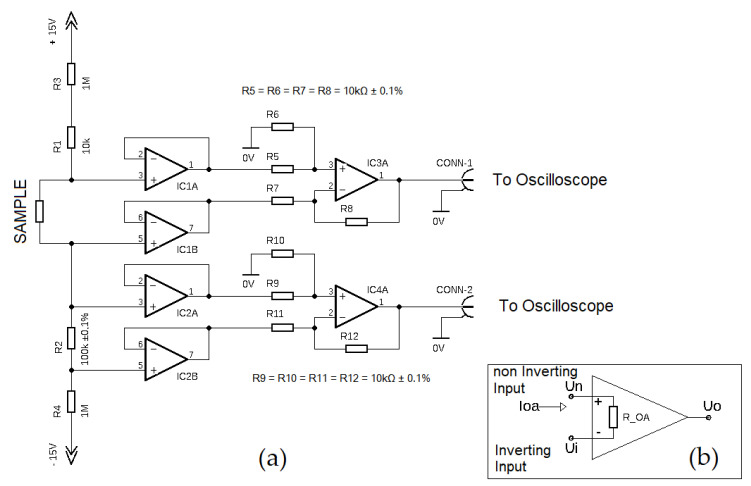
(**a**) The electric scheme of measurement; (**b**) operational amplifier.

The experimental setup for DC EER measurement is shown in Figure 3.

The measuring was based on the indirect method, whereas the complete measuring setup was compiled and assembled for this study by the authors. The resistance was calculated from voltage drop on the measured sample and voltage drop on the serial high precision resistor *R*_2_ (Figure 3a). Since all changes in the load were slow, a simple Ohm’s Law was used in the form

(1) $$R_{S}=U_{S}/I_{S},$$

where $R_{S}$ is the resistance of the sample (Figure 3a), $U_{S}$ is the voltage drop across the sample (measured directly) and $I_{S}$ is the current going through the sample (measured indirectly from the voltage on the $R_{2}$).

Operational amplifiers (OA) assured the sensibility of measurements. Directly connected OA (denoted in the Figure 3a as IC1A, IC1B, IC2A, IC2B) were used as voltage followers. For OA functionality explanation, a simplified scheme is depicted in Figure 3b. Following the theory of the real OA output voltage, it can be assumed that

(2) $$U_{o}=A_{u}\left(U_{n}-U_{i}\right),$$

where, $U_{o}$ is the output voltage, $U_{i}$ is the inverting input voltage, $U_{n}$ is the non-inverting input voltage, and $A_{u}$ is the open loop voltage amplifying coefficient.

Theoretically, the difference $\left(U_{n}-U_{i}\right)$ can be assigned as $U_{dif}$ If the OA is connected as the voltage follower, then the inverting input voltage $U_{i}$ is equal to the output voltage $U_{o}$. Equation (1) will then be modified with mathematical adjustment to the form

(3) $$U_{o}=U_{n}A_{u}/\left(A_{u}+1\right)$$

where $A_{u}$ is the open loop voltage amplifying coefficient. $A_{u}=100000$ typically, for a used amplifier. Due to this condition, $U_{o}$ can be taken as equal to the $U_{n}$. The input current passing through OA can be written as

(4) $$I_{OA}=U_{dif}/R_{OA}$$

and $R_{OA}$ is internal resistance of OA.

Equation (4) can be modified with the substitution from Equation (2) and reads as follows:

(5) $$I_{OA}=U_{0}/\left(R_{OA}\times A_{u}\right)$$

Substituting the value of *U*_0_ from Equation (3) in Equation (5) leads to the framing of Equation (6):

(6) $$I_{OA}=U_{n}/\left(R_{OA}\times \left(A_{u}+1\right)\right)$$

where the input resistance is transformed by multiplication with the term ($A_{u}+1$). So, taking into consideration the characteristic value of internal resistance for used OA (the minimal value *R_OA_* = 30 kΩ), the customized installation is able to easily measure the resistances up to 750 MΩ and even higher. This fact guarantees that measuring method is suitable for tested samples.

Amplifiers IC3A, IC4A joined behind voltage followers were connected as typical differential amplifiers with voltage magnification [26,27]. Data recording was obtained using digital multichannel oscilloscope Rigol MS05104 (Rigol Technologies, Co Ltd., Suzhou, China). 

Digital Image Correlation (DIC) was applied to determine the local strain fields in the studied samples. For this purpose, a stochastic pattern made by an anti-reflex spray, MR2000 Anti-Reflex L (MR Chemie GmbH, Unna, Germany) was applied on the surface of all the tested samples. The strains of the complete sample were recorded over the testing protocol via CCD monochrome camera Baeumer PXU 60 M Q (Bauemer, Frauenfeld, Switzerland) with a sampling frequency of 15 Hz. The DIC process was controlled over the software GOM Snap 2D, (GOM, Braunschweig, Germany). Subsequently, the captured pictures were processed and analyzed with DIC software (GOM Correlate, Braunschweig, Germany) for the strain field evaluation.

## 3. Results and Discussions

### 3.1. Local Strain Distribution and Measured EER

**Figure 4:**
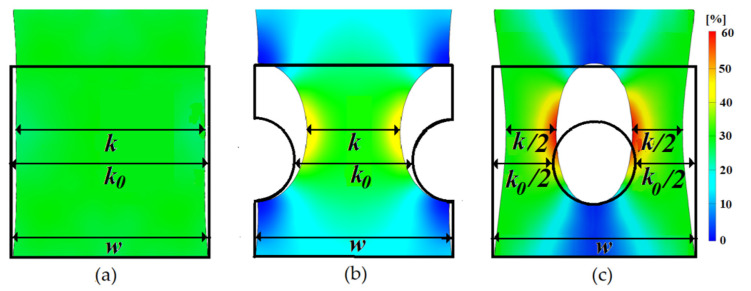
Strain maps (**a**) sample type a, (**b**) sample type b and (**c**) sample type c.

The measured strain contour image for all the studied samples under tensile loading and their initial shapes are visualized in Figure 4. Due to inappreciable differences in the determined local strains between the rubber samples loaded with different concentrations of CB, only the rubber samples compounded with 70 phr CB will be discussed in terms of DIC characterization. The DIC software monitored the deformation of complete sample during straining and evaluated the strain over the complete sample surface, as well as the local strain near to inhomogeneity and presented the data in colored map over the complete surface of the sample.

For all the analyzed samples, the contraction was observed in the horizontal axis, orthogonally to the main strain. The contraction gradually decreased in the vertical direction from the horizontal axis, whereas close to the clamping area, the contraction became minimal. It is obvious that the location of the inhomogeneity in the edges (sample type b) and central part (sample type c) provoked a very different strain distribution across the horizontal axis as well as over the complete affected sample area due to the stress concentrations focused on the sharp corners due to the abrupt change in the surface area [28].

**Figure 5:**
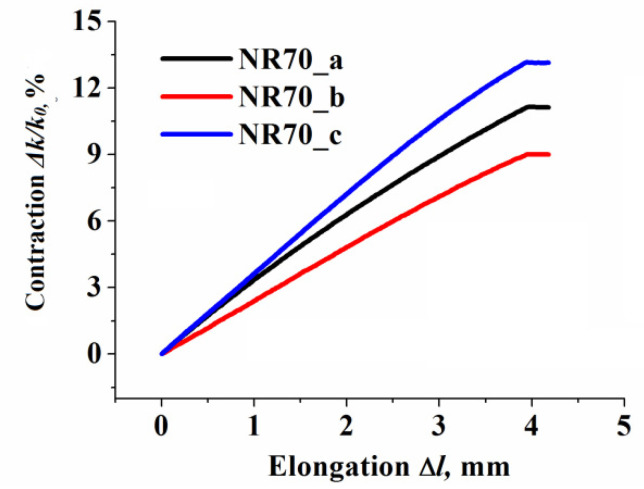
The evolution of elongation vs. contraction for sample containing 70 phr of CB (similar trends were observed for all tested CB concentrations).

The DIC of dynamic evolution of the contraction of the sample ($\Delta k=k_{0}-k$) during a testing protocol was monitored continuously by measuring the width of the sample passing through the geometrical center (see Figure 4). To avoid any misunderstanding, here and in all following text, *k* represented the width of rubber composite in the geometrical center of sample, which is not always equal with samples width (w), due to intentionally create inhomogeneity. The vertical displacement of the sample (elongation, noted as Δ*l*) was proportional to applied strain, and fully depended on the settings of the tensile equipment. However, the horizontal contraction of the monitored segment was a material and also the sample shape dependent term. The results experienced a similar trend for all CB concentration. Thus, only those obtained for the sample containing 70 phr CB are presented in Figure 5. Sample type (a) exhibited an absolutely logical trend of contraction, in close agreement with Poison’s ratio value (ν = 0.5 for CB filled natural rubber). For samples containing geometrical inhomogeneity, the elongation–contraction relationship exhibited an appreciable difference due to complicated non-homogeneous local strain distribution.

**Figure 6:**
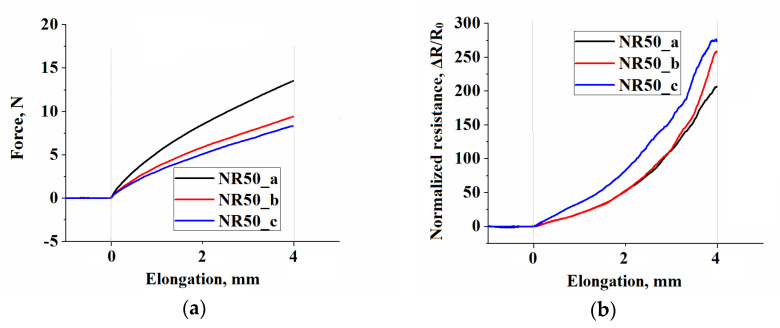

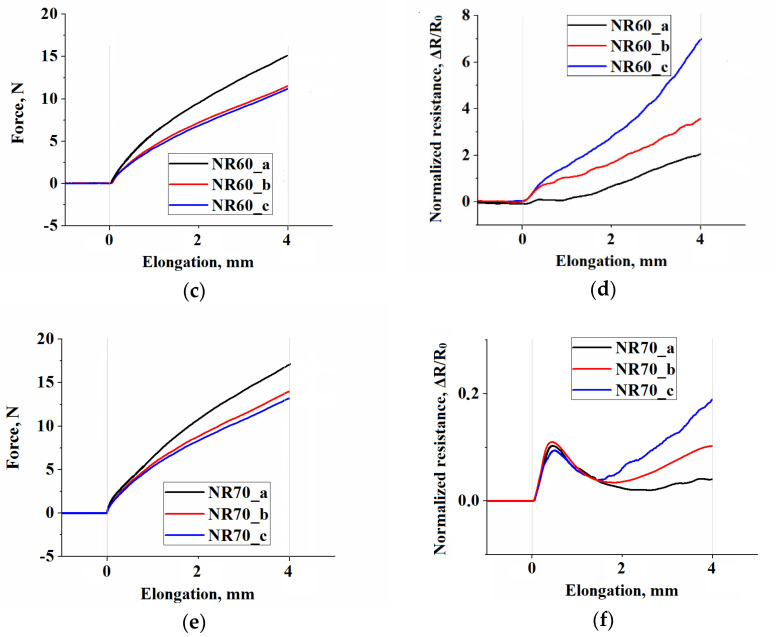
(**a**,**c**,**e**) force registered during the testing protocol; (**b**,**d**,**f**) electrical resistance of samples.

The representative curves of resulting force and EER vs. elongation corresponding to applied strain, for all different filler concentrations and three different sample types are shown in Figure 6. From the mechanical point of view, the plot of force vs. elongation (Figure 6a,c,e) shows the well-known and expected trend of increasing force with an increase in the filler content [29] over the complete tensile loading.

**Figure 7:**
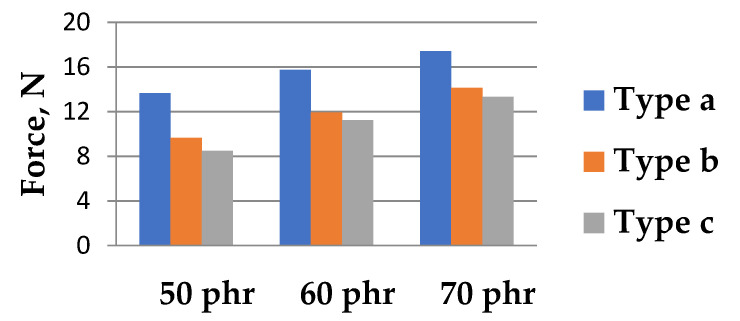
Force at maximum set elongation in strained samples.

The two samples containing different positions of geometrical inhomogeneity, although having the same total initial cross-sectional area in the horizontal axis, revealed a difference in the measured force under a certain strain over the complete straining process. The forces developed by tensile machine to achieve the maximum set strain are depicted in Figure 7. The sample type b possessing an undivided cross-section area exhibited higher force if compare to sample type c having a divided one. The cause of observed difference was the variation in the location of produced inhomogeneity, resulting in a different new surface area creation, preserving the same total cross-sectional area of the two different samples. Therefore, the sample with bulk cross-section area (type b) required more energy to be deformed compared to the divided one (type c) due to an excess in the number of internal molecular bonds.

Figure 6b,d,f exhibit the uniqueness of the results of EER for the used deformation settings. A gradual increase in CB concentration increased the non-monotonic runway of resistance variation. The results are presented in the form of a ratio between variation of resistance (Δ*R* = *R* − *R*_0_) and *R_0_*, the latter being the resistance of the sample before deformation. Samples containing 50 phr of CB (Figure 6b) exhibited progressive, well-distinguished increase in resistance as a function of applied strain for all the geometries of the samples. Depending on sample geometry, increase in samples resistance (Δ*R*/*R*_0_) attained an impressive value close to 270 times at the maximum applied strain. This fact is highly appreciated for sensitivity of sensors.

Samples containing 60 phr CB (Figure 6d) showed an essential difference of Δ*R*/*R*_0_ in dependence of sample geometry, exhibited a modest increase in resistance for about seven times in the best case, while the samples containing 70 phr of CB (Figure 6f) showed a very low Δ*R*/*R*_0_ ranging between 0.02 and 0.2 times for maximum applied stress.

### 3.2. Current Propagation Mode Switching Effect

The most unusual results were obtained for samples filled with 70 phr CB (Figure 6f). In just one tensile event, an initial increase in resistance was followed by a sudden decrease and then again, a continuous increase. Sequentially, such a strange behavior may be regarded as a negative and positive piezoresistive effect in filled polymers, discussed earlier, for example, in [30]. The uniqueness of the observed behavior was to have both these phenomena in the same material and under one loading process. This effect has not been discussed in any previous scientific studies from theoretical as well as an experimental point of view. In the opinion of the authors of the present article, the explanations given in the subsequent discussion describe the observed peculiarities in the effective resistance variation of the sample under deformation:

The contraction of the sample during the tensile test (Figure 4) confirmed the concurrence of two processes taking place in the conductive network. In the direction of applied tensile strain, the distance between the conductive particles increased and contrary to this happening, in the perpendicular direction of the applied strain, a hydrostatic pressure, made the particles approach each other. CB particles are usually considered as spheres coupled into aggregates. Taking into consideration the Hertzian contact theory, the contact area, *A*, of two spheres of identic radius, R, can be calculated from the equation:

(7) $$A=\pi \frac{R}{2}\times x$$

where *x* is the penetration depth between spheres.

The Poisson coefficient, *ν*, for thin sample of square shape exhibits the ratio between contraction, Δ*k*, and its elongation, Δ*l*, and following expression can be written:

(8) $$\Delta l=\frac{\Delta k}{\nu }.$$

**Figure 8:**
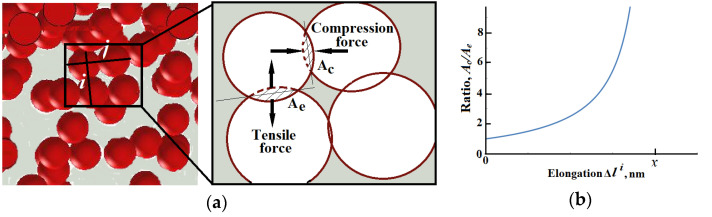
(**a**) Scheme of contact areas conductivity model; (**b**) Ratio between parallel and perpendicular contact area vs. interparticle displacement in tensile direction.

Theoretically, the analysis of the evolution of contact areas, *A_e_* and *A_c_* which represent the contact area between two arbitrary carbon black spheres incorporated in rubber matrix, coaxially aligned, perpendicular to the direction of strain and to direction of contraction, respectively. Taking into consideration that $\Delta l^{i}$ is the elongation between two particles forming couple *i*, and $\Delta k^{j}$ is the contraction between particles forming couple *j* (see Figure 8a),

(9) $$A_{c}=\pi \frac{R}{2}\times \left(x+\Delta l^{i}\right)\;\mathrm{and}$$

(10) $$A_{e}=\pi \frac{R}{2}\times \left(x-\Delta k^{j}\right).$$

Thus, the ratio

(11) $$\frac{A_{c}}{A_{e}}=\frac{x+\Delta l^{i}}{x-\Delta k^{j}}=\frac{x+\Delta l^{i}}{x-\nu \cdot \Delta l^{i}}$$

The solutions to this equation are depicted in Figure 8b.

Considering the framework of the present model, the relative motion of the CB particles in accordance to the Poisson’s ratio of a rubber composite, the applied strain resulting in interparticle displacements $\Delta l^{i}$ will generate, as well, an interparticle contraction $\Delta k^{j}$, proportional to *ν*. The aforementioned solution to Equation (11) clearly delimitates two regions: $\Delta l^{i}<x$ where the contact area of particles aligned in the direction of strain decrease much slower than the increase in the contact area of particles aligned in contraction direction, in effect, generating a global increase in interparticle contact area with possibility for easier carriers’ flow (visualization sketch in Figure 8a). With further increase in strain, the increase of interparticle displacement becomes larger than the penetration depth ($\Delta l^{i}\geq x$), signalizing the moment when the particles lose their contact and the current propagation change its mechanism, switching from Ohmic to Shottki or trap-assisted tunneling mechanisms. Since the EER is proportional to contact area, it was assumed that this phenomenon was the reason behind the sudden increase in the conductivity followed by a moderate decrease during the deformation of the sample. A reasonable question— “Why is this effect not observed for samples containing 50 or 60 phr CB?”—could be answered by assuming the initially predominant charge transfer mechanism to be the tunneling one.

### 3.3. The Deformed Samples EER Estimation

**Figure 9:**
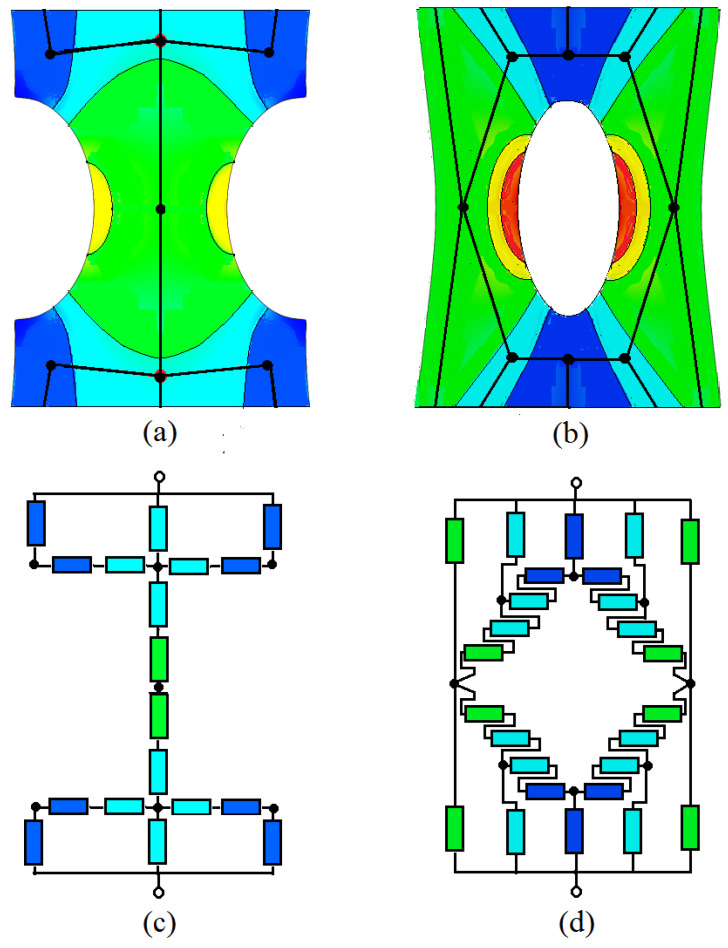
(**a**) Sketch defining the resistive domains with corresponding nodes for samples type b and (**b**) for samples type c, (**c**) The equivalent circuit for samples type b and (**d**) equivalent circuit for samples type c.

The aim of this paragraph was to check the possibility of EER calculation of non-homogeneously deformed samples by simulation, which will help to solve and thus replace a vital problem for otherwise finding the electrical properties of a product by cost and time demanding direct experimental testing. DIC converted the macroscopic domains of deformed samples into the colored maps, symbolizing the different magnitude of endured strain and consequently the difference in resistivity. As a base for present EER estimations it served the studies dedicated to effective resistance of homogeneously distributed two or three phase containing materials, these cases presenting with big approximations the conditions created in deformed materials. The main concept was founded on discrete networks of resistors, possessing one mutual node in each discrete domain and connecting the middle of the neighbor domain border [7]. Thus, the effective resistance calculation of deformed samples was reduced to calculation of equivalent circuit resistance (Figure 9). Taking into consideration the Kirchhoff’s rules, and principles of symmetries, the contribution of maximally strained domains (yellow and red colored) was neglected assuming that current will not flow through these domains.

For simplification, the resistivity of each domain was considered homogenous and isotropic (contrary to the findings in the previous paragraph). Thus, the domain resistance was divided into equal resistances connecting the node of the domain with neighboring domains. Generally, the resistance of one rectangular domain may be calculated applying the formula:

(12) $$R=\rho \frac{l}{d\cdot w}.$$

where *R* is the total resistance of the analyzed domain; ρ is its resistivity; *l*, *d* and *w* are the length, thickness and width respectively.

**Figure 10:**
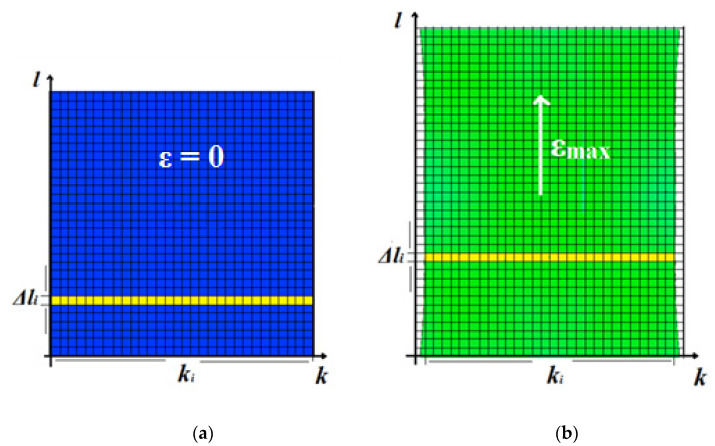
Illustration for resistance calculation for samples of arbitrary shape: (**a**) undeformed and (**b**) deformed state.

For the non-rectangular (arbitrary) shapes of samples (a usual characteristic of deformed samples), Equation (12) can be rewritten as:

(13) R=ρd∑i=1lΔlΔliΔki

where $\Delta l_{i}$ is an arbitrarily chosen length of cell for an imaginary sample partition, *i* is the position of the cell along the tensile direction, *d* is the thickness of the sample considered in the present work as a constant, and $\Delta w_{i}$ is the width of the sample in position *i* (see Figure 10). For successful calculation, it remained to find the resistivity of domains in accordance to experienced local strain depicted in different colors on the DIC map. It should be noted that only the reference samples (type a) exhibited a homogeneous strain during the whole testing protocol, giving reason to consider the resistivity of the deformed samples as well homogeneous.

An approximate resistivity of domains (color delimited) was determined by extrapolation of fitted curve of referential samples EER (type a from Figure 6b,d,e) and subsequent conversion into resistivity. In the case of samples loaded with 70 phr of CB, the part of the curve containing the switching effect was omitted for the fitting process. The results of extrapolation are presented in Figure 10.

As can be observed, the sample containing 70 phr CB exhibited a reverse trend compared with the samples with lower filler content.

**Figure 11:**
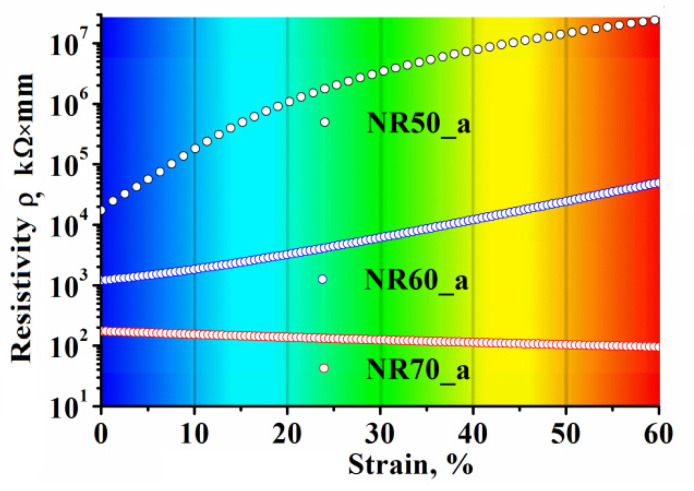
The resistivity of reference samples containing 50 phr CB, 60 phr CB and 70 phr CB vs. the sample’s strain and corresponding DIC domain’s color.

Finding the resistivity of each domain from Figure 11 according to color, dictated by local strain (shown in Figure 9) and applying Equation (13), resistance of the separate domains—the constituent parts of deformed at maximum strain samples types b and c—was calculated (see Table 2 and Table 3).

Finally, the calculated resistance of each domain was evenly divided to the corresponding number of imaginary resistors to fulfill the equivalent circuit (Figure 9), where the colors of the resistors denote their belonging to respective resistive domains. The rule of resistive domain partition into resistors was described earlier, in the introductory part. Thus, the complicated task to calculate the EER of non-homogeneously deformed sample was reduced to calculation of total resistance of an ordinary resistors network.

**Figure 12:**
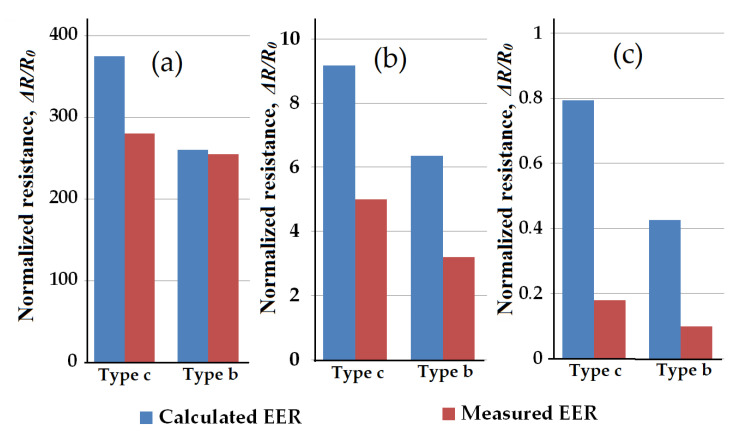
The relative effective electric resistance for samples type b and c experimentally measured and calculated: (**a**) samples containing 50 phr CB, (**b**) 60 phr CB and (**c**) 70 phr CB.

The results of calculated EER compared with the measured ones for sample types b and c determined at maximal applied strain, are presented in Figure 12. It is clearly visible that qualitatively the calculated data follows the identical trends observed during experimental investigation, for all sample types and CB loading.

Taking into consideration the accepted approximations the method of EER of non-homogeneously deformed samples by discrete resistors network approach, showed a reasonably good accuracy, and could be considered as a prospective one for related studies.

## 4. Conclusions

In the present study, the effects of filler loading at 50, 60 and 70 phr CB in NR matrix on variation in strain induced electric resistance was investigated. At such comparatively high filler loadings, a nonlinear strain-resistance variation was observed which in general is a huge impediment for stress–strain self-sensing applications. The obtained results showed a strong limitation of strain induced resistance growth pronounced with increasing filler concentration. As such, samples containing 60 phr and 70 phr of CB could be considered as non-efficient for strain gauge fabrication. However, this work was not only confined to prove this inefficiency and was further channelized to investigate some other interesting phenomena.

Thus, in continuation, a very first trial for theoretical EER calculation of non-homogeneously deformed samples was done. Initially, by fitting and extrapolation of experimental results for a rectangular reference samples (considered homogeneously strained) the strain-dependent resistivity of studied compounds was found. Using this strain-dependent resistivity and further, following the discrete resistors network approach with the simultaneous application of DIC technique to determine locally strained domains, reasonably good calculated EER results were obtained for non-homogeneously deformed samples. This finding opens a large possibility for practical application through simulation of EER for arbitrary shaped products under deformation.

Even with some differences between the magnitudes of the calculated and measured results, the trends were the same and thus, the proposed method may “feel” the geometrical inhomogeneities and their locations. For all concentrations of CB, it was found that the placement position of inhomogeneity has a tremendous impact on local strain distribution as well as on the EER. This relation could be the base for in-situ, nondestructive defect monitoring technology.

Finally, a unique current propagation mode switching phenomenon was observed and explained in a novel approach. The trustable explanation of this effect was done by analyzing the simultaneous decrease in the contact area of the conductive CB particles in the direction of strain and an increase in the perpendicular contraction direction. This effect, according to the knowledge of the authors of the present work was not previously reported.

## Figures and Tables

**Table 1:** Rubber formulation.

	NR	Oil	Carbon Black	CBS	Sulfur	ZnO	Stearic Acid
	Content in phr *						
**NR50_#**	100.00	10.00	50.00	1.00	2.50	5.00	2.00
**NR60_#**			60.00				
**NR70_#**			70.00				

*—parts per hundred of rubber by weight. #—a, b or c depending on sample geometry (see Figure 1).

**Table 2:** The calculated resistivity and resistance of separate domains (samples type b).

		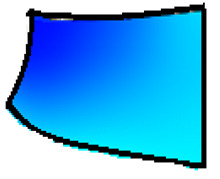	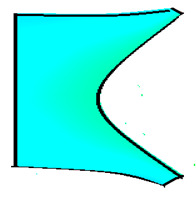	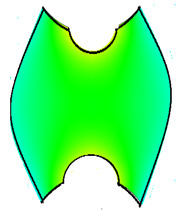	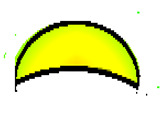
**50 phr**	***ρ*, Ωm**	6.80 × 10^4^	8.10 × 10^5^	3.60 × 10^6^	9.20 × 10^6^
	***R*, Ω**	9.50 × 10^7^	3.10 × 10^8^	3.10 × 10^9^	3.10 × 10^10^
**60 phr**	***ρ*, Ωm**	1.40 × 10^3^	2.80 × 10^3^	6.00 × 10^3^	1.40 × 10^4^
	***R*, Ω**	1.90 × 10^6^	1.00 × 10^6^	5.10 × 10^6^	4.60 × 10^7^
**70 phr**	*ρ*, Ωm	1.70 × 10^2^	1.40 × 10^2^	1.20 × 10^2^	1.10 × 10^2^
	***R*, Ω**	2.40 × 10^2^	5.30 × 10^1^	1.10 × 10^2^	3.60 × 10^2^

**Table 3:** The calculated resistivity and resistance of separate domains (samples type c).

		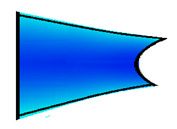	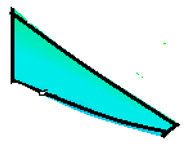	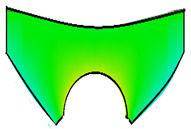	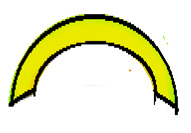	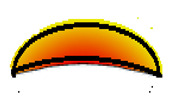
**50 phr**	***ρ*, Ωm**	6.80 × 10^4^	8.10 × 10^5^	3.60 × 10^6^	9.20 × 10^6^	2.10 × 10^7^
	***R*, Ω**	3.40 × 10^7^	1.80 × 10^9^	6.30 × 10^9^	5.20 × 10^10^	4.90 × 10^10^
**60 phr**	***ρ*, Ωm**	1.40 × 10^3^	2.80 × 10^3^	6.00 × 10^3^	1.40 × 10^4^	3.90 × 10^4^
	***R*, Ω**	7.00 × 10^5^	6.10 × 10^6^	1.10 × 10^7^	7.90 × 10^7^	9.30 × 10^7^
**70 phr**	***ρ*, Ωm**	1.70 × 10^2^	1.40 × 10^2^	1.20 × 10^2^	1.10 × 10^2^	9.80 × 10^1^
	***R*, Ω**	8.80 × 10^4^	3.10 × 10^5^	2.10 × 10^5^	6.30 × 10^5^	2.30 × 10^5^

## Data Availability

The data presented in this study are available on request from thecorresponding author.

## Nomenclature

*A_c_* (nm^2^)	Contact area between CB particles, aligned perpendicular to the direction of sample contraction.
*A_e_* (nm^2^)	Contact area between CB particles, aligned perpendicular to the direction of sample strain
*A_u_* (-)	Open loop voltage amplifying coefficient
*d* (mm)	Thickness of sample
Δ*k* (mm)	Contraction of sample
$\Delta k^{j}$ (nm)	The contraction between two particles forming couple *j*
Δ*l* (mm)	Elongation of sample
$\Delta l^{i}$ (nm)	The elongation between two particles forming couple *i*
$\Delta l_{i}$ (mm)	Length of cell *i* for an imaginary sample partition
Δ*R*/*R*_0_ (-)	Normalized resistance
$\Delta w_{i}$ (mm)	The width of the sample in position *i*
$I_{OA}$ (A)	The input current passing through operational amplifier
*I_s_* (A)	Current going through the sample
*l* (mm)	Length of sample
*ν* (-)	Poisson’s ratio
*R_s_* (Ω)	Resistance of the sample
*R_OA_* (Ω)	Internal resistance of operational amplifier
ρ (Ωm)	Resistivity
*U_s_* (V)	Voltage drop across the sample
*U_i_* (V)	Inverting input voltage
*U_o_* (V)	Output voltage
*w* (mm)	Width of sample
*x* (nm)	The penetration depth between two arbitrary CB particles

## Abbreviations

AC	Alternating current
CB	Carbon black
CBS	N-cyclohexyl-2-benzothiazolesulfenamide
DIC	Digital Image Correlation
DC	Direct current
EER	Effective electric resistance
EMT	Effective medium theory
ERN	Equivalent resistor network
HAF	High abrasion furnace
NR	Natural rubber
OA	Operational amplifier
phr	Parts per hundred rubber
SIC	Strain induced crystallization
ZnO	Zinc oxide
